# The complete chloroplast genome of a coastal plant, *Euphorbia jolkinii* (Euphorbiaceae)

**DOI:** 10.1080/23802359.2022.2055502

**Published:** 2022-04-03

**Authors:** Hiroyuki Iwata, Takuro Ito, Goro Kokubugata, Koji Takayama

**Affiliations:** a Department of Botany, Faculty of Science, Kyoto University, Kyoto, Japan; b Botanical Gardens, Tohoku University, Sendai, Japan; c Department of Botany, National Museum of Nature and Science, Tsukuba, Japan; d Department of Botany, Graduate School of Science, Kyoto University, Kyoto, Japan

**Keywords:** Coastal plant, chloroplast genome, *Euphorbiaceae*, *Euphorbia jolkinii*, genetic structure

## Abstract

The complete chloroplast genome sequence of a coastal plant, *Euphorbia jolkinii* Boiss. (Euphorbiaceae), was determined. The chloroplast genome was 162,854 bp in length, consisting of a large single copy region (90,726 bp), a small single copy region (18,422 bp), and two inverted repeats (26,853 bp). The chloroplast genome contained 115 genes, consisting of 80 unique protein-coding genes, 30 unique tRNA genes, four unique rRNA genes, and one pseudogene, *rps16*. GC content of the whole chloroplast genome was 35.6%. The phylogenetic analysis showed a close relationship between *E. jolkinii* and *E. pekinensis* Rupr. The sequence data would provide useful information to understand the evolutionary process of *E. jolkinii*.

The genus, *Euphorbia* L., is a gigantic genus of angiosperm, consists of approximately 1900 species, and has a cosmopolitan distribution mainly in tropic to temperate regions (Mabberley 2008). Some *Euphorbia* species well adapt to arid environments. *Euphorbia jolkinii* Boiss. 1860, which is the focus of this study, has widely but patchily distributed on the coastal regions in Taiwan, South Korea, and Japan (Kurosawa 1999). Ocean currents convey their seeds, and it may maintain gene flow among the geographically isolated populations of *E. jolkinii*. However, the phylogeography and genetic structure of the species have not been evaluated well. Complete chloroplast genomes would provide useful information to understand gene flow patterns by seed dispersal because of their maternal nature inheritance in many angiosperms. In this study, we determined the complete chloroplast genome sequence of *E. jolkinii*.

Leaf sample of *E. jolkinii* was collected from Tomogashima Island, Wakayama Prefecture, Japan (N34.2855, E135.0186). The voucher specimen of the collected sample was deposited in the herbarium of Kyoto University, KYO (https://www.museum.kyoto-u.ac.jp, Hidetoshi Nagamasu, nagamasu@inet.museum.kyoto-u.ac.jp, the voucher number is Iwata & Takayama 18060401). Total DNA was extracted using Plant Genomic DNA Extraction Mini Kit (Favorgen Biotech Corp., Ping-Tung, Taiwan). DNA library was prepared using Lotus DNA Library Prep Kit and xGen Stubby Adapter with Unique Dual Index Primer Pairs (Integrated Device Technology, Inc., San Jose, CA) according to the manufacturer's instructions. The DNA library was sequenced by the Illumina Hiseq platform at Macrogen Japan (Tokyo, Japan). Approximately 52 million 150 bp raw pair-end reads were obtained. Low-quality nucleotides and reads, and reads containing adapter sequences were removed by Trimmomatic 0.39 (Bolger et al. 2014). The chloroplast genome was assembled using GetOrganelle pipeline (Jin et al. 2020). GeSeq in CHLOROBOX web service was used for the annotation of the chloroplast genome (Tillich et al. 2017). The chloroplast genome sequence and annotation were submitted to DDBJ (LC661698).

The complete chloroplast genome sequence of a coastal plant *E. jolkinii* (Euphorbiaceae) was 162,854 bp in length, consisting of a large single copy region (90,726 bp), a small single copy region (18,422 bp), and two inverted repeats (26,853 bp). The chloroplast genome contained 115 genes, consisting of 80 unique protein-coding genes, 30 unique tRNA genes, four unique rRNA genes, and one pseudogene, *rps16*. The *rps16* gene was found to lose function due to the insertion of stop codons and large modifications of amino acid sequences. The overall GC content of the cp genome was 35.6%, and that of large single copy region, small single copy region, and inverted repeats was 32.8%, 29.6%, and 42.3%, respectively.

The length of the complete chloroplast genome sequences of *Euphorbia* species ranged from 159,466 bp to 164,340 bp. The number of annotated genes in the chloroplast genome was slightly different among the species, but the order of the protein-coding genes was consistent.

We constructed a phylogenetic tree based on the chloroplast genome sequences of *E. jolkinii*, other 13 *Euphorbia* species (14 accessions), and *Balakata baccata* (Roxb.) Esser 1999 as the outgroup. All the cp genome data were aligned with MAFFT version 7.450 (Katoh and Standley 2013). Modeltest-NG (Darriba et al. 2020) was used to find the best fitting model. Maximum-likelihood tree was constructed under GTR + GAMMA + I model by RAxML-NG (Kozlov et al. 2019). Bootstrap values were calculated with 1000 replicates. The phylogenetic analysis indicated that *E. jolkinii* was closely related to *E. pekinensis* Rupr. 1859 with 100% bootstrap support (Figure 1). Further, the three species, *E. jolkinii*, *E. pekinensis*, and *E. helioscopia* L. 1753 from *Euphorbia* section *Helioscopia*, used in this study formed a monophyletic clade with 100% bootstrap support. *Euphorbia jolkinii* might have newly adapted to coastal environments in their speciation process and maintained their wide distribution range by their sea-drifted seed dispersals. The information of the complete chloroplast genomes will shed light on the evolutionary process and phylogeography of the species.

**Figure 1.**
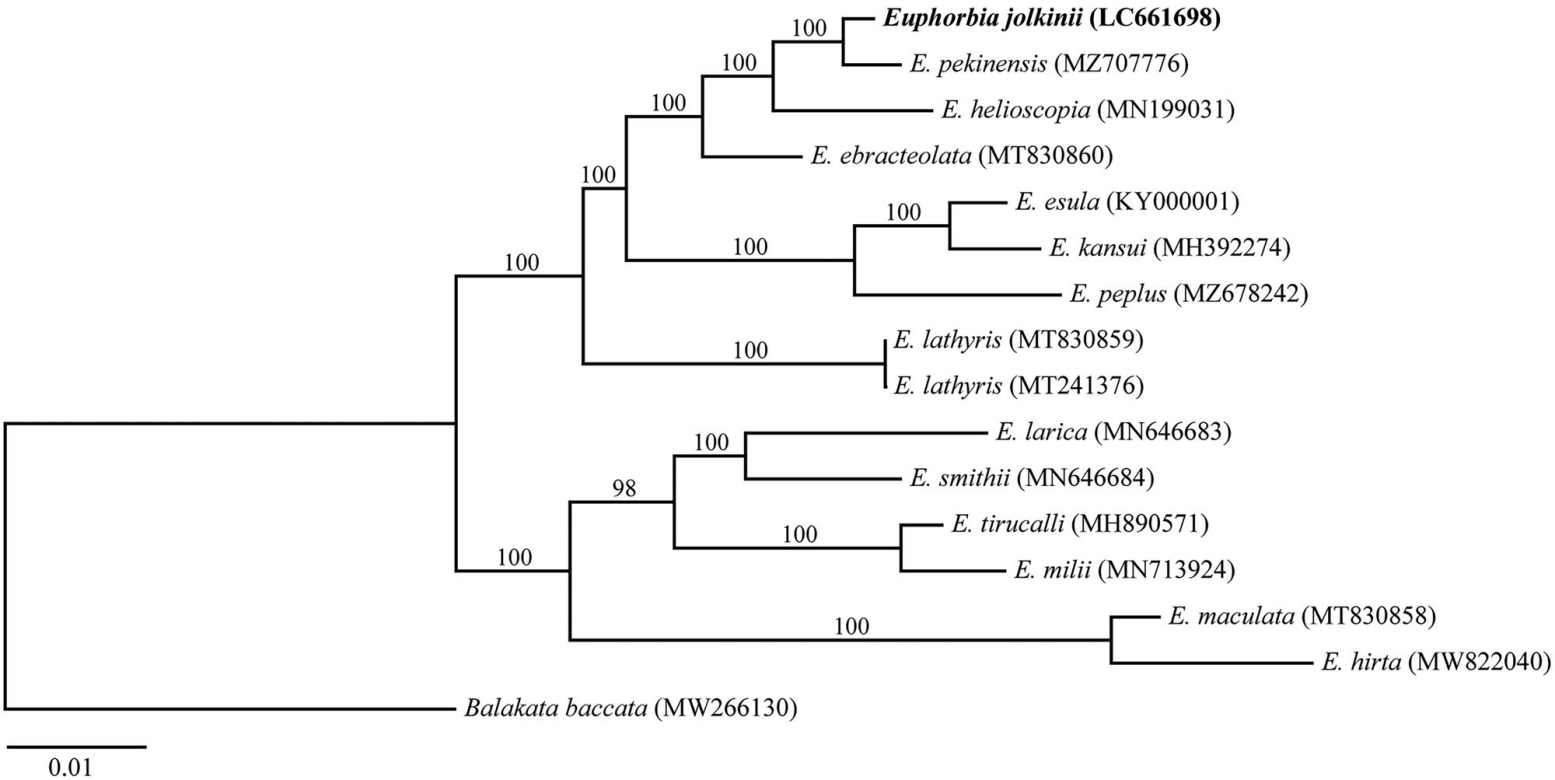
The maximum-likelihood tree based on 15 complete chloroplast genomes of *Euphorbia* and one outgroup species. The bootstrap value was shown on each branch.

## Data Availability

Data that support the findings of this study are openly available in DDBJ (DNA Data Bank of Japan) and can be accessed at http://getentry.ddbj.nig.ac.jp/top-e.html; Accession number LC661698. The associated BioProject, BioSample, and SRA numbers are PRJDB12950, SAMD00441096, and DRR344859, respectively.

## Plant collecting permission statement

Plant materials were collected under the permission from the Ministory of the Environment Gaverment of Japan (Kinki number 2006032).
